# Integration in Working Memory: A Magnetic Stimulation Study on the Role of Left Anterior Prefrontal Cortex

**DOI:** 10.1371/journal.pone.0043731

**Published:** 2012-08-24

**Authors:** Nicola De Pisapia, Marco Sandrini, Todd S. Braver, Luigi Cattaneo

**Affiliations:** 1 CIMeC - Center for Mind/Brain Sciences, University of Trento, Rovereto (TN), Italy; 2 DiSCoF – Department of Cognitive Science and Education, University of Trento, Rovereto (TN), Italy; 3 Center for Neuroscience and Regenerative Medicine, Uniformed Services University of the Health Sciences, Henry M. Jackson Foundation, Bethesda, Maryland, United States of America; 4 Human Cortical Physiology and Stroke Neuro-Rehabilitation Section, National Institute of Neurological Disorders and Stroke, National Institutes of Health, Bethesda, Maryland, United States of America; 5 Cognitive Control and Psychopathology (CCP) Laboratory, Department of Psychology, Washington University in St. Louis, St. Louis, Missouri, United States of America; Ecole Normale Supérieure, France

## Abstract

Integration is a fundamental working memory operation, requiring the insertion of information from one task into the execution of another concurrent task. Previous neuroimaging studies have suggested the involvement of left anterior prefrontal cortex (L-aPFC) in relation to working memory integration demands, increasing during presentation of information to be integrated (loading), throughout its maintenance during a secondary task, up to the integration step, and then decreasing afterward (unloading). Here we used short bursts of 5 Hz repetitive Transcranic Magnetic Stimulation (rTMS) to modulate L-aPFC activity and to assess its causal role in integration. During experimental blocks, rTMS was applied (N = 10) over L-aPFC or vertex (control site) at different time-points of a task involving integration of a preloaded digit into a sequence of arithmetical steps, and contrasted with a closely matched task without integration demand (segregation). When rTMS was applied during the loading phase, reaction times during secondary task were faster, without significant changes in error rates. RTMS instead worsened performance when applied during information unloading. In contrast, no effects were observed when rTMS was applied during the other phases of integration, or during the segregation condition. These results confirm the hypothesis that L-aPFC is causally and selectively involved in the integration of information in working memory. They additionally suggest that pre-integration loading and post-integration unloading of information involving this area may be active and resource-consuming processes.

## Introduction

In this study we examined the role of the left anterior prefrontal cortex (L-aPFC) in a high-level cognitive function: the integration of information held in working memory. Working memory [1] refers to the ability to temporarily hold and manipulate cognitive information during the execution of goal oriented tasks. Working memory operations include maintaining, updating, inhibiting and transforming mental content in accordance with task requirements. Working memory tasks are known to engage several brain regions, in particular frontal areas are more involved in manipulation of information [2], [3], whereas posterior regions (temporal and parietal, depending on the stimulus modality) are more involved in maintenance of information [4], [5]. These brain-behavior relationships have been confirmed by previous Transcranial Magnetic Stimulation (TMS) studies during working memory tasks [6], [7].

One particular working memory operation that in the last years has received special attention is the coordination and integration of information during concurrent task execution. Mental arithmetic is a domain in which such operations are particularly required. For instance, in sequences like: (7×(15−6)+ …), the working memory steps required are: (a) to mentally load 7, (b) to compute (15–6 = 9) while concurrently maintaining 7 in mind, (c) to integrate the partial result from step b with the loaded number (7×9 = 63), (d) to unload 7 (now irrelevant), and continue on with the computations (63+ …). Early studies [8], [9], [10], [11], [12], [13], [14] have consistently found that integration operations involve the anterior part of the prefrontal cortex. This vast cortical region is particularly developed in humans compared to other primates, and converging consensus indicates that it is critical in enabling flexible cognitive control and management of multiple mental tasks [15], [16]. The integration of separate information into ongoing working memory processing is not exclusive to mental arithmetic, but it has also been shown to take place in a variety of cognitive domains, such as episodic memory [17], language [18], logical deduction [19], and analogical reasoning [12]. Nevertheless, the extent to which all these types of integration between concurrent processes involve the same anterior prefrontal areas across domains is a matter of current research [20].

**Figure 1:**
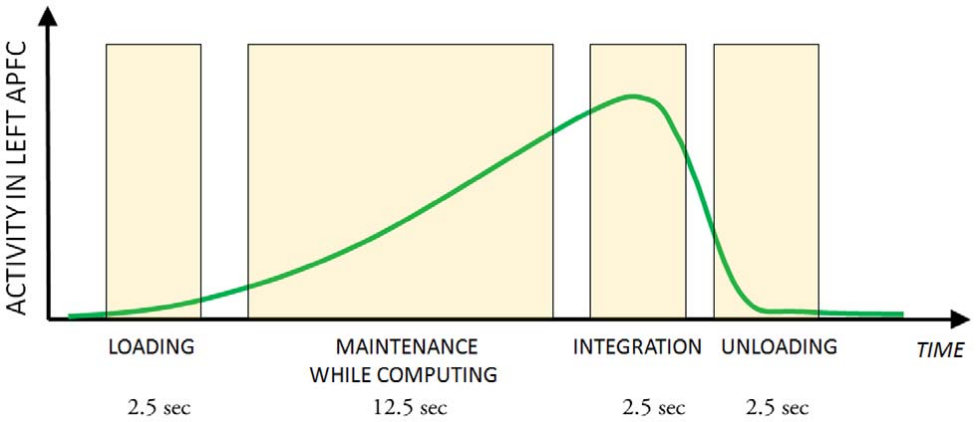
Activity in L-aPFC. A schematic representation of activity in L-aPFC during an integration task, derived from previous fMRI studies; durations refer to the various phases of the experimental design [21], [22].

In previous functional Magnetic Resonance Imaging (fMRI) studies [21], [22], we focused on the integration operation during mental arithmetic tasks. Mental arithmetic operations require integration quite frequently, and previous experiments [23], [24] have shown that arithmetic integration implies unique working memory resources. We found that a specific L-aPFC region was active when volunteers were required to maintain information from a primary task consisting in encoding a digit (the *preload*), which then they were required to integrate into a secondary task consisting in executing consecutive arithmetic steps (similarly to the previous arithmetic example). L-aPFC activity started to grow from preload presentation up to the integration step, but then decayed back to baseline quickly after integration was completed (see Fig. 1).

In another experimental condition of the original fMRI studies, volunteers performed a very similar assignment, also involving the encoding of an initial digit and the subsequent sequence of arithmetical computations, but the two tasks were kept segregated all along, and there was no integration requirement (i.e., the preload was simply recalled at the end of the secondary task, and not inserted into the arithmetic computations). In this segregation condition, L-aPFC showed no specific involvement, and thus we argued that its main selective role was in preparing for the integration operation. One key interpretation of the particular activity dynamics of L-aPFC that we observed in the prior studies (Fig. 1), and that we wanted to test in the current study, is that this region is specialized for the maintenance of information in the outer loop of a nested hierarchy, while an inner loop is constantly updating. Thus, the role of this prefrontal region is to load outer loop information, preserve it from the updating process taking place in the inner loop, and make it available to other regions that actually implement the integration step per se, and finally release it after it has been utilized. Such outer-inner loop interpretation is coherent with computational analyses of prefrontal controlling mechanisms in working memory processing [25], and also with prominent recent studies by Koechlin and colleagues [8], [16] proposing that anterior prefrontal cortex is involved in tasks requiring “cognitive branching”, consisting in the maintenance of primary task goals (i.e., the outer loop) while concurrently allocating attention to subgoals (i.e., the inner loop). In our framework, a further necessary requirement to specifically activate L-aPFC is that outer loop information is linked to information that at a certain point will have to be integrated into the inner loop processing. In our studies, it was only the integration condition that activated L-aPFC, and not the segregation condition, even though both involved outer loop information that was maintained during inner loop processing. Yet in the segregation condition, the two loops were kept separated and there was no requirement to integrate information. Thus, our interpretation was that the involvement of this specific region in working memory is linked to implementing an intention in the middle of an ongoing subtask, and not just in maintaining two concurrent task goals.

The aim of the current study was to use TMS as a convergent cognitive neuroscience methodology to gather additional information regarding the functional role and specialization of L-aPFC during working memory integration. In particular, we tested whether TMS-based exogenous modulation of L-aPFC excitability influenced task performance specifically during conditions and time-points that we propose involve such integration operations.

According to our previous fMRI studies, activity in L-aPFC during integration trials (see Fig. 1) goes through four successive phases: (1) it starts at preload presentation; (2) it ramps up during the simultaneous math calculations and maintenance of preload information; (3) it reaches a peak during the integration step; (4) it decays after the integration step. Contrary to previous TMS studies in working memory, that focused more on posterior (i.e., dorsolateral) prefrontal cortical regions [6], [26], in the present experiment we applied time-locked repetitive TMS (rTMS) to modulate L-aPFC excitability and to assess its causal role in integration. RTMS was applied over L-aPFC or vertex (control site) at different time-points of a task involving integration of a preloaded digit into a sequence of arithmetical steps, and contrasted with a closely matched task except for the integration demand (segregation). Specifically, we used an on-line rTMS protocol with short high frequency (5 Hz) bursts that were delivered in several experimental conditions during the four phases of the integration operation in working memory. We chose 5 Hz rTMS because previous studies successfully modulated working memory using this stimulation frequency [27], [28]. In addition, this frequency is a good compromise between the advantages of high-frequency TMS, and the possibility to keep the number of pulses very low in order to reduce the discomfort induced by rTMS over this brain area. Our main goals were to show that: (a) L-aPFC has a causal role in integration; (b) L-aPFC has no causal role in segregation; (c) L-aPFC activity acts in a preliminary fashion, that is to say it starts at preload presentation. Additionally, by varying the specific timing of rTMS delivery, our goal was to explore L-aPFC engagement in the various phases of integration.

## Materials and Methods

### Participants

This study was approved by the University of Trento Ethical Committee. It included sixteen healthy participants, carefully screened for contraindications to rTMS [29]. All of them expressed their written informed consent to all the procedures.

### Procedure

**Figure 2:**
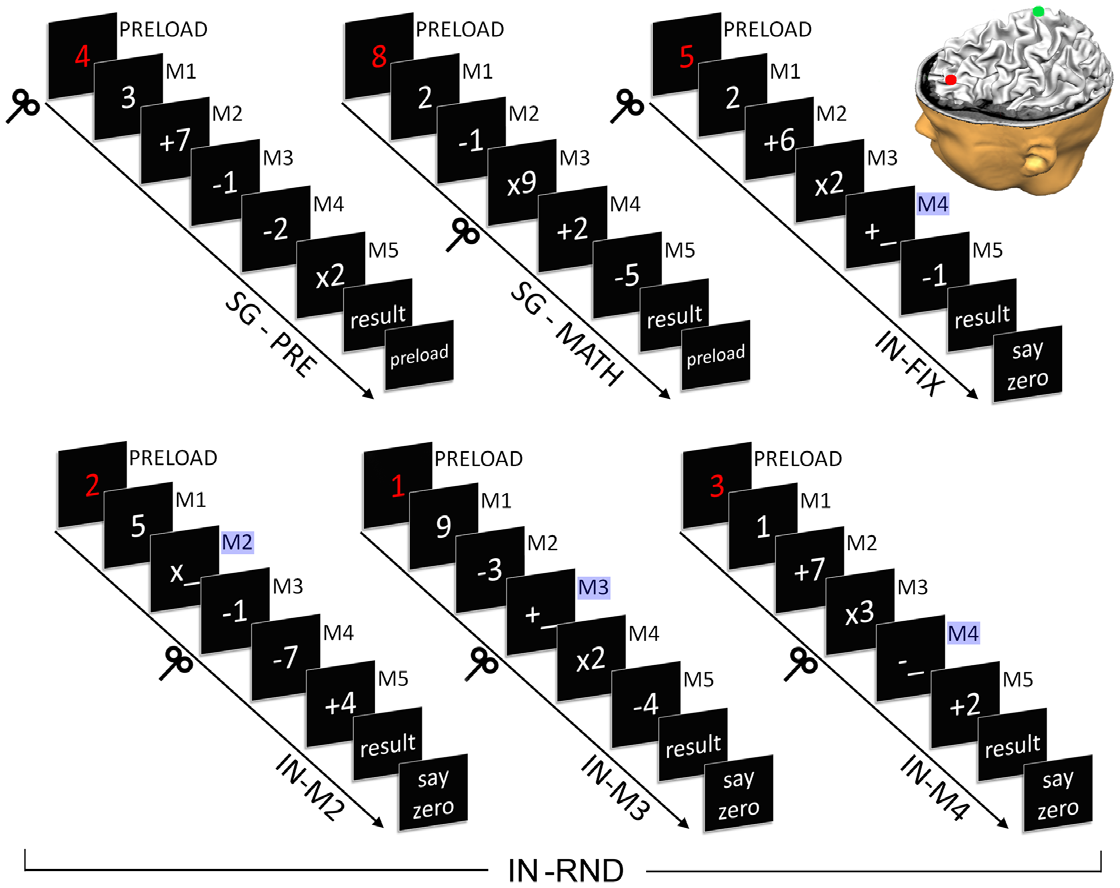
Experimental design. In all conditions, volunteers first saw a digit (preload, in red), which they had to keep in mind (primary task). This was followed by a sequence of arithmetic computations (secondary task; M1 to M5). In all steps, volunteers were required to press a button as quickly as possible whenever they had completed the specific request. In two segregation conditions (SG-PRE and SG-MATH), after M5 they had to state verbally the result of the arithmetical computations, and then the preload digit. RTMS was delivered (symbolized by a TMS coil in the figure) at the beginning of preload presentation in SG-PRE trials or at the beginning of step M3 in SG-MATH trials. In two integration conditions (IN-FIX and IN-RND), volunteers instead were required to mentally insert the preload into the ongoing computations (integration steps colored in blue in the figure). This happened predictably at step M4 in IN-FIX blocks (upper right of figure), or unpredictably in IN-RND blocks (three trial types at bottom of figure). Unpredictable integration could take place at steps M3, M4 or M5, accounting for the three trial types (IN-M3, IN-M4, IN-M5). In all integration trials, after M5, participants had to state vocally the result, and finally to utter the digit “zero” (to match the design of the segregation conditions). RTMS was delivered during preload in IN-FIX, and always at M3 during any of the 3 IN-RND trial types, thus allowing the investigation of rTMS effects before (IN-M4), during (IN-M3) and after (IN-M2) the integration step. RTMS was applied in two distinct sites in different blocks: the target area (L-aPFC, red spot in the inset) and a control area (vertex, green spot in the inset).

Participants were tested while seated comfortably, with head movements minimized by a chinrest. Single trials are schematized in Fig. 2, and they involved the following sequence of events. First, a visually presented single digit (colored in red) appeared in the first frame (PRELOAD). Next, a five-step mental arithmetic problem was sequentially presented in a frame-by-frame manner. Each of the five steps of the trial (designated as M1 to M5) consisted of a single-digit (1 to 9, colored in white) and a mathematical operator (also in white, they could be +,−, or ×). Digits and operators were selected randomly, with the constraint that in no step the total could go below 0 or above 50. In the segregation conditions (SG), the preload information had to be actively maintained but separated from the mental arithmetic task. In the integration conditions, the preload had to be mentally inserted into the mental arithmetic problem at a specified step. There were two types of integration conditions; one block with fixed conditions (IN-FIX), in which the integration step was constant across trials (M4), and three blocks with random conditions (IN-RND), in which the integration step was unpredictably varied across trials, namely at step M2 (IN-M2), at step M3 (IN-M3) or step M4 (IN-M4).

After a short practice in each of the block types (6 trials each), single experimental trials of the same type were presented in blocks of 12. Participants were warned at the beginning of each block whether they were going to perform a SG, IN-FIX or IN-RND (in this last case, without further specification of when the integration step was to occur). Six blocks were carried out in association with rTMS applied over L-aPFC, and six other blocks, with the same trial composition as the previous 6 blocks were carried out in association with rTMS applied over the vertex (Cz) calculated according to the 10–20 EEG system, which served as control stimulation site.

The 6 block types for each of the two TMS conditions were: 2 segregation (SG-PRE: rTMS during preload; SG-MATH: rTMS during M3); four integration (IN-FIX: rTMS at preload; 3 IN-RND blocks with rTMS at M3, each with an equal proportion of IN-M2, IN-M3 and IN-M4 trials, such that the total number of these trial-types matched the number of trials in the other block types, i.e. 12 each). The order of the blocks was randomly intermixed between subjects. The order of TMS site was counterbalanced, such that two consecutive subjects had a different order in the sequence of TMS targets (Cz or L-aPFC) in each block type. In sum, in the whole experiment every subject had 12 repetitions of each of the 6 trial types, in each of two TMS conditions.

For all conditions, each trial frame was presented until button press, or for a maximum time of 2 seconds (response window). In case of no response within this maximum time, the task advanced to the next frame; in case of response, a fixation cross was shown during the remaining time of the response window. The stimulus-onset asynchrony (SOA) between trials was always 2.5 seconds, thus allowing for a minimum 500 msec interval between trials (also with a fixation cross). Volunteers were instructed to complete encoding or arithmetic computations as quickly as possible and then indicate completion of each task with a button press (space-bar on computer keyboard). Two volunteers whose accuracy was below 40% in at least 2 conditions were not included in the analyses. Stimuli were presented using E-Prime 2.0. Reaction times (RTs) were recorded with a button box connected to the computer used for stimuli presentation. Verbal responses were recorded through a microphone and subsequently re-played to analyze accuracy during preload recall (segregation) and arithmetical computations (all conditions).

### Neuronavigation

For each subject a structural MRI scan was acquired before the experiment to be used for MRI-neuronavigated positioning of the TMS coil. A high-resolution T1-weighted magnetization-prepared rapid gradient echo sequence (176 axial slices, in-plane resolution 256 3 224, 1-mm isotropic voxels, generalized autocalibrating partially parallel acquisition with acceleration factor = 2, time repetition = 2200 msec, time echo = 4.180 msec, time to inversion = 1020 msec, flip angle = 7°) scan of the brain of each subject was obtained using a MedSpec 4-T head scanner (Bruker BioSpin GmbH, Rheinstetten, Germany) with an 8-channel array head coil.

Starting from this scan, a 3D reconstruction of the scalp and brain surfaces was produced using the BrainVoyager software (Brain Innovation BV, The Netherlands). The 3D brain surface was then transformed into Talairach space in order to identify in individual subjects the cortical spot corresponding to coordinate x = −33 y = 42 z = 21, derived from a previous functional imaging study involving similar tasks [21]. The targeted area was therefore in the left middle frontal gyrus, between Brodmann areas 46 and 10.

Once the target location of the L-aPFC region was identified, the brain surface was re-transformed in native proportions in the AC-PC (Anterior Commissure – Posterior Commissure) space for neuronavigation. The BrainVoyager neuronavigation software combined with an ultrasound tracking system (Zebris Medical GmbH, Isny, Germany) was used to coregister the 3D scalp reconstruction with the actual participant’s head and thus marking the target point for rTMS on the real head.

### Magnetic Stimulation

Biphasic TMS pulses were applied using a figure-of-eight coil (MC-B70) and a MagPro 3100 stimulator (MagVenture A/S, Denmark). Right before the experiment, the individual resting motor threshold was defined as the lowest stimulation intensity applied over the primary motor cortex capable of evoking a visible contraction in the relaxed right hand in at least 5 out of 10 consecutive stimuli. The stimulation intensity for the experiment was set to 95% of the individual threshold in order to reduce the discomfort induced by rTMS. A train of 4 pulses was delivered at a frequency of 5 Hz (600 msec duration) simultaneously with stimulus presentation of the specific frame (thus there was a total of 48 pulses in each block, and a total of 576 pulses for each participant). In case of vertex stimulation, the coil was oriented in line with the longitudinal fissure and the coil handle pointed posterior. In case of L-aPFC, the coil handle was angled backwards at about 45° away from the midline and its correct positioning was repeatedly checked.

### Statistical Analysis

An analysis of RTs was conducted on trials in all experimental conditions. RTs of trials in which there was an error either on preload recall (for segregation conditions) or on math result (for all conditions) were not included in the analyses. Six separate ANOVAs were conducted for each of the six experimental modalities (SG-PRE, SG-FIX, IN-FIX, IN-M2, IN-M3 and IN-M4). In each 6×2 ANOVA the dependent variable was the participant’s mean RT and the independent variables were two within-subjects factors: ‘step’ (6 levels: from PRELOAD to M5) and ‘TMS’ (two levels: L-aPFC or vertex). The verbal steps of the trial (last two) were omitted from calculation, since participants were not required to respond as fast as possible in those particular steps. We opted for fragmenting the analysis between the 6 experimental conditions rather than performing an omnibus 6×6×2 ANOVA, because the different steps numbered from 1 to 6 were not comparable between tasks and therefore they did not entirely fulfill the rationale for considering them a within-subjects repeated measure. Indeed, aside from the ordinality of their sequence, the six different steps of the segregation (SG-PRE and SG-MATH), predictable integration (IN-FIX) and unpredictable integration (IN-RND) conditions involve different underlying cognitive processes. On the contrary, the assumption for considering each step a repeated within-subject measure was met in the 3 IN-RND conditions, which thus in a subsequent step we compared directly in a single ANOVA. To do so we performed a repeated measures ANOVA that combined the 3 IN-RND conditions, and further utilized a more focused temporal window of interest consisting of steps M3 and M4, i.e. the step in which rTMS was applied and the following one, thus we performed a 3 (integration conditions)×2 (TMS conditions)×2 (steps) repeated measures ANOVA. POST-hoc tests were carried out for all ANOVAs with Tukey’s Honestly Significant Difference (HSD) tests as calculated by the STATISTICA 8.0 software (Statsoft Inc.).

An analysis of error rates was performed between the two TMS conditions, using a series of t-tests for paired data. These were calculated as the ratio between the number of arithmetic sequences that ended with a wrong result and the total number of sequences (n = ***12***) within an experimental modality.

## Results

Four participants did not complete the experiment due to local discomfort after a few TMS applications to the frontal area; two other participants were excluded from the analyses because their accuracy was very low (percentage error higher than 40% in at least two conditions). The remaining 10 participants experienced no discomfort from TMS and were able to perform the task smoothly and without interruptions. No immediate or delayed adverse side effects were reported.

**Figure 3:**
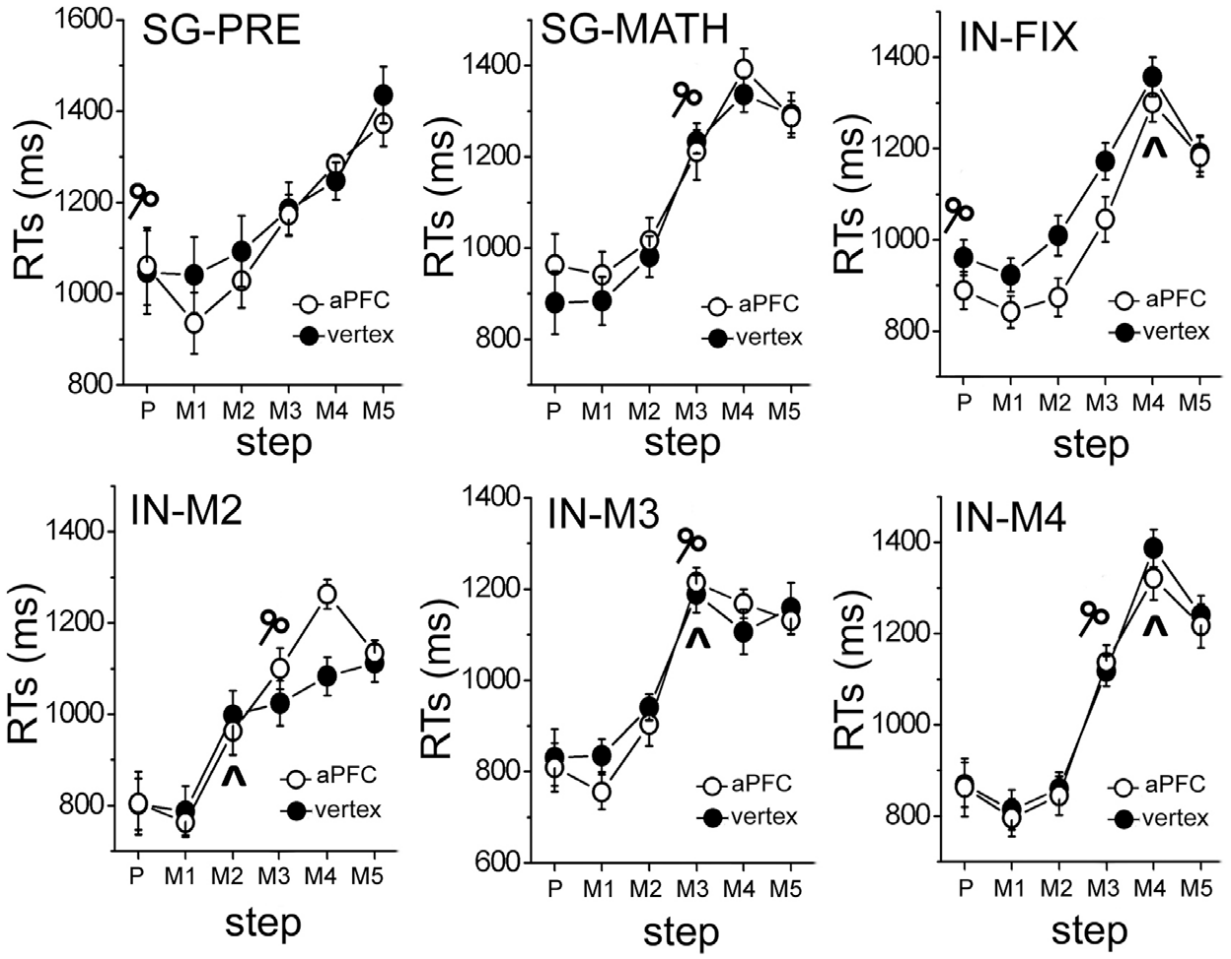
Behavioral data. RTs in the different conditions and in the frames from preload presentation (P in the figure) to M5, both for when targeting the vertex (black dots) and L-aPFC (white dots). The schematic coil represents time of rTMS delivery, and the caret symbol (∧) indicates the integration step. Error bars are standard errors of the mean.

In Fig. 3 we report average RTs for the button presses indicating completion of calculation: the schematic coil represent time-occurrence of rTMS delivery, the caret symbol (∧) indicates integration steps (during integration blocks only), the black dots correspond to RTs with rTMS at vertex, and white dots correspond to RTs with rTMS at L-aPFC. Notice that the delivery of the rTMS pulses always at step M-3 during IN-RND blocks allowed us to investigate the effects on subtask execution when integration occurred before (IN-M2), during (IN-M3) and after (IN-M4) rTMS delivery.

All 6 of the ANOVAs on RTs showed significant results of the ‘step’ factor (all p-values <0.000001). More interestingly, in two conditions we found a significant effect of the ‘TMS’ factor. In condition IN-FIX a significant main effect of ‘TMS’ was found (F(1, 9) = 6.49, p = 0.031), reflecting that RTs for the aPFC stimulation trials were significantly faster than those for the vertex stimulation trials (1022 msec vs. 1102 msec). In the IN-M2 condition we found a significant interaction of the ‘TMS’ and ‘step’ factors (F(5,45) = 2.49, p = 0.04). Post hoc comparisons showed that only RTs of step 5 (M4) were significantly different between TMS conditions (p = 0.04), while RTs in other steps were not (all p values >0.93).

In the 3×2×2 repeated measures ANOVA that combined the 3 IN-RND conditions (3 integration conditions ×2 TMS conditions ×2 steps) we found a significant interaction between the ‘integration’ and ‘TMS’ factor (F(2, 18) = 5.17 p = 0.02).

Finally, we focused on the IN-M2 condition, and paired t-tests comparisons between the RTs in the two TMS conditions at each single step showed a significant difference between them only at step M4 (p = 0.02, corrected), as in the previous analysis; in addition, we found that all other effects or interactions were non-significant (minimum p-value = 0.29). No significant effects were seen in t-tests on error rates. Table 1 shows the mean error rates and standard deviations in all conditions.

**Table 1:** Error rates (standard deviations in parenthesis) for recall of preload (upper part) and for mental arithmetic computations (lower part) in the various experimental conditions, divided for when rTMS pulses were delivered to the vertex (control) or to the target area (L-aPFC).

PRELOAD RECALL	CONTROL	L-aPFC
SG-PRE	18.333% (11.653)	17.5% (15.441)
SG-MATH	13.333% (11.249)	11.667% (16.292)
**MENTAL ARITHMETIC**	**CONTROL**	**L-aPFC**
SG-PRE	19.167% (9.6625)	21.667% (17.656)
SG-MATH	34.167% (19.425)	25% (19.245)
INT-FIX	15% (9.4608)	11.667% (9.7816)
IN-M2	11.667% (9.7816)	9.1667% (6.1489)
IN-M3	14.167% (11.146)	13.333% (9.7816)
IN-M4	18.333% (12.91)	20.833% (13.176)

No significant effects were seen in t-tests on error rates.

## Discussion

In this rTMS study we aimed at investigating whether (a) a portion of the L-aPFC is causally involved in integration, (b) its involvement occurs from the time that to-be-integrated information is loaded into working memory until the time of unloading, (c) it is not involved in segregation. Additionally, we aimed at investigating whether it is selectively involved during the various phases of the integration operation in working memory. The comparison between performance in a segregation condition (concurrent tasks with no integration requirements) when targeting L-aPFC clearly showed no difference in performance, independently of whether rTMS was delivered simultaneously with preload (SG-PRE) or during the arithmetical computations (SG-MATH), compared to when targeting a control area (vertex). On the contrary, during integration conditions we found that rTMS could facilitate or interfere with performance depending on the exact time of delivery and on the underling cognitive process taking place, thus confirming its causal role specifically in the integration operation. In particular, we found that rTMS: (1) facilitated performance when delivered during the loading phase (IN-FIX), (2) interfered with performance when delivered during the unloading phase (IN-M2), and (3) had no effect when delivered during maintenance while computing the mathematical steps (IN-M4) or when delivered during the integration step itself (IN-M3).

The results of this study support the hypothesis derived from our previous neuroimaging studies [21], [22] that L-aPFC acts like a specialized buffer, which enables sustained maintenance, in an outer loop, of information that will subsequently need to be integrated into the on-going processing of a concurrent inner loop (which might take place in posterior prefrontal regions). The role of L-aPFC may thus be of loading, preserving and finally unloading outer-loop information during inner-loop processing, but selectively when the cognitive system expects an upcoming step of integration of information, given that we observed no L-aPFC involvement during segregation conditions.

A buffering component of working memory involved with integration (the episodic buffer) has been theorized in the past [30], but prior accounts have not postulated a preparatory function, which we instead found to be characteristic of this area. In the current experiment, we found further evidence of the causal involvement of L-aPFC activity in preparation for integration (i.e., starting at preload presentation), but not during closely matched segregation conditions requiring concurrent execution of mental arithmetic tasks without integration demands.

**Figure 4:**
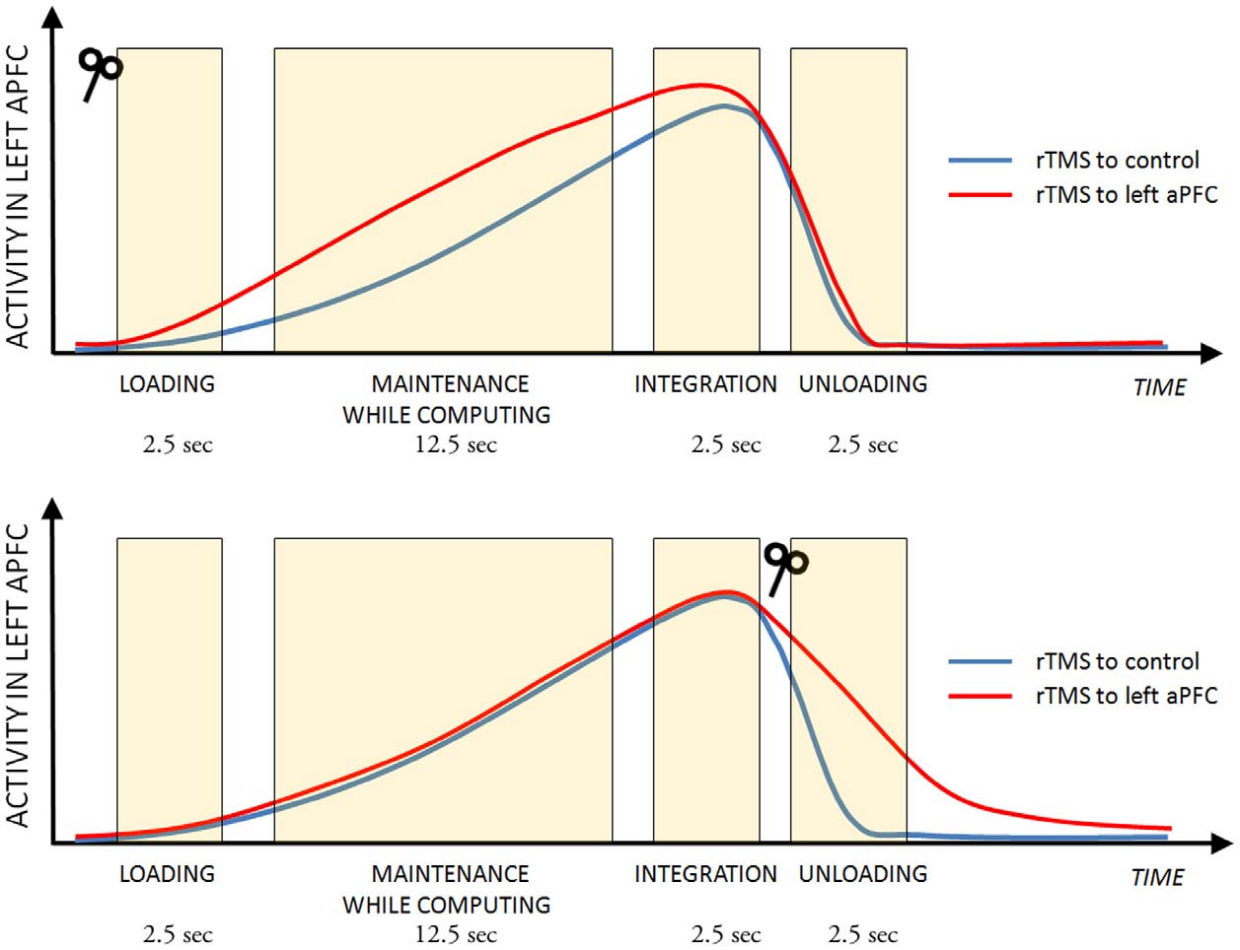
Effects of rTMS on L-aPFC. Schematic representation of rTMS effects on activity in L-aPFC when this area is targeted during the various phases of integration, in comparison with control rTMS (targeting the vertex). The durations reported refer to the various phases of the experimental design. This figure is only to illustrate our interpretation, and it does not represent actual data. The TMS-coil symbols indicate time of rTMS delivery. The hypothesized sustained L-aPFC activity from the loading phase up to integration and the subsequent fading during unloading are directly derived from the results of two previous neuroimaging studies (Fig. 1) [21], [22]. Faster RTs in IN-FIX after rTMS to L-aPFC before loading can be tentatively explained with increased activity in this area inducing stronger control over maintenance of preload information in the face of interference coming from secondary task processing (top panel). Slower RTs in IN-M2 after rTMS to L-aPFC during unloading could be explained with increased activity which induces a difficulty in discharging preload information, which becomes irrelevant after integration has taken place. Of course, further experiments (e.g., including the simultaneous use of TMS and functional neuroimaging recordings) would be needed to validate these hypotheses.

Regarding the various phases of the integration operation, these results support the hypothesis of a primary causal role of L-aPFC in the preparation for integration, given the facilitation on RTs observed with rTMS during preload presentation (IN-FIX). Additionally, the interference effects observed with rTMS when unloading (IN-M2) suggest instead that rTMS stimulation increased the difficulty of properly processing the release of information after it has been utilized (see a schematic representation of our interpretation in Fig. 4).

Notably, loading and unloading in integration are phases in which L-aPFC is updating information, contrary to the other phases in which it is mainly implementing maintenance operations. Prominent neurocomputational models support the view that lateral prefrontal regions might be critically involved in working memory updating functions, either via interactions with bottom-up signals arising from the basal ganglia [25], [31], or from phasic dopamine release [32], or by exerting a top-down excitatory bias on information that is maintained elsewhere (i.e., parietal cortex) [33]. We observed that the delivery of rTMS during information maintenance (IN-M4) or the integration step itself (IN-M3) did not produce any behavioral effects. These results tentatively suggest that rTMS did not modulate activity in the absence of afferent updating signals; but other experiments (for example, by adopting concurrent TMS and fMRI) are necessary to more clearly interpret these findings concerning the specific phases of integration.

In this study we found a full spectrum of TMS effects (facilitation, neutral and interference). Most TMS studies predict changes in behavior, but – as in our case - they are rarely explicit about their direction (facilitation or interference). The direction depends upon a number of variables, such as intensity, duration, frequency, pattern of pulses applied, the initial neural activation state of the targeted region and the cognitive task being performed [34]. In on-line paradigms, these effects can be either facilitatory or disruptive depending on the time point of stimulation [35]. For example, TMS usually disrupts cognitive functions when applied during the time period in which the stimulated area contributes to the task, but a number of studies have reported facilitation effects when TMS is delivered early in the time course of a trial – before activation in the region was expected [36], [37], [38], [39]. When TMS is delivered before the onset of a cognitive process –as in our study - neural populations are less influenced by central factors than when TMS is applied during a cognitive process. Although the difference in the initial neural activation state could explain why the effects of TMS are opposite in the two circumstances, further studies are necessary to corroborate this hypothesis [40]. Furthermore, the prior interpretations of TMS effects have referred to TMS applied to cortical areas that are strictly task-relevant. In contrast, for the current experiment, the target area is instead presumably involved only in part of the task (preparation for integration), and thus it is difficult to predict how rTMS to that region can affect performance.

Finally, another alternative interpretation to consider is whether the observed effects in this experiment could be attributed to the fact that rTMS applied to the most anterior frontal areas might produce eye blinking. Eye blinking can be induced by TMS over frontal regions by two separate mechanisms. The first is reflex blinking, as part of a trigeminofacial reflex [41]. This produces bilateral eyelid closure peaking around 80 msec after stimulation [42], which could indeed interfere with stimulus perception. One argument against this hypothesis is that reflex blinking habituates almost completely after one single repetition of trigeminal stimulation with 200 msec inter-stimulus intervals [43]. Also, this hypothesis would lead to the prediction that performance is worse in all cases of stimulation in the exact step in which it was applied. In our case not only we found a spectrum of inhibitory (IN-M2), neutral (SG-PRE, SG-MATH, IN-M3 and IN-M4) or facilitative (IN-FIX) effects due to prefrontal stimulation, but we also observed effects that, irrespective of polarity, lasted for several seconds (IN-M2 and IN-FIX) after the time of stimulation, which is definitely not compatible with a simple peripheral occlusion of vision related to eyelid closure. The second mechanism by which frontal rTMS can produce blinking is a direct stimulation of the ramus orbitalis of the facial nerve on the left side. This phenomenon does not habituate for repeated stimuli, but can only account for closure of one eyelid only (the left), therefore leaving practically unaltered the flow of visual of information to the central nervous system. In short, the pattern of results in the current study are not well-explained by the alternative explanation of TMS-induced blinking phenomena.

### Conclusion

The present study supports the view that L-aPFC is (a) causally involved in the integration process in working memory, (b) its involvement lasts from the time preload presentation up to the integration step, and therefore seems to be related to anticipation of the integration step, (c) it is not involved during segregation. The results indicate that rTMS in L-aPFC could modulate performance during phases of integration in which working memory contents are updated with information from the primary task (i.e., both loading and unloading of this information), and not during the phases in which the information had to be stably maintained during secondary task processing, or during the point of integration itself. As such, the findings suggest are consistent with, and thus indicate the need to further investigate, the hypothesis that L-aPFC plays a functional role in working memory integration, but potentially in a surprising way. It seems that this region contributes to updating processes that enable both preparation for integration and resetting of working memory after integration is completed, but seems to be less involved in the act of integration *per se*.
